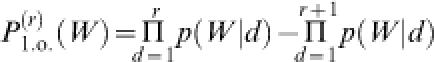# Experimental Rugged Fitness Landscape in Protein Sequence Space

**DOI:** 10.1371/journal.pone.0000096

**Published:** 2006-12-20

**Authors:** Yuuki Hayashi, Takuyo Aita, Hitoshi Toyota, Yuzuru Husimi, Itaru Urabe, Tetsuya Yomo

**Affiliations:** 1 Department of Bioinformatic Engineering, Osaka University, Suita, Osaka, Japan; 2 Rational Evolutionary Design of Advanced Biomolecules (REDS) Group/JST, Saitama Small Enterprise Promotion Corporation SKIP City, Kawaguchi, Saitama, Japan; 3 Department of Functional Materials Science, Saitama University, Saitama, Japan; 4 Department of Biotechnology, Osaka University, Suita, Osaka, Japan; 5 Graduate School of Frontier Biosciences, Osaka University, Suita, Osaka, Japan; 6 Expoloratory Research for Advanced Technology (ERATO), Japan Science and Technology Agency (JST), Suita, Osaka, Japan; Fred Hutchinson Cancer Research Center, United States of America

## Abstract

The fitness landscape in sequence space determines the process of biomolecular evolution. To plot the fitness landscape of protein function, we carried out *in vitro* molecular evolution beginning with a defective fd phage carrying a random polypeptide of 139 amino acids in place of the g3p minor coat protein D2 domain, which is essential for phage infection. After 20 cycles of random substitution at sites 12–130 of the initial random polypeptide and selection for infectivity, the selected phage showed a 1.7×10^4^-fold increase in infectivity, defined as the number of infected cells per ml of phage suspension. Fitness was defined as the logarithm of infectivity, and we analyzed (1) the dependence of stationary fitness on library size, which increased gradually, and (2) the time course of changes in fitness in transitional phases, based on an original theory regarding the evolutionary dynamics in Kauffman's *n*-*k* fitness landscape model. In the landscape model, single mutations at single sites among *n* sites affect the contribution of *k* other sites to fitness. Based on the results of these analyses, *k* was estimated to be 18–24. According to the estimated parameters, the landscape was plotted as a smooth surface up to a relative fitness of 0.4 of the global peak, whereas the landscape had a highly rugged surface with many local peaks above this relative fitness value. Based on the landscapes of these two different surfaces, it appears possible for adaptive walks with only random substitutions to climb with relative ease up to the middle region of the fitness landscape from any primordial or random sequence, whereas an enormous range of sequence diversity is required to climb further up the rugged surface above the middle region.

## Introduction

*In vitro* molecular evolution can be considered an adaptive walk on a fitness landscape in sequence space, where “fitness” is a quantitative measure of a certain physicochemical property of a biopolymer, such as thermostability or enzymatic activity [1], [2]. The “fitness landscape” is a map of the fitness of each sequence into the corresponding point in the sequence space, and the “adaptive walk” consists of evolutionary changes in the sequences on the fitness landscape. The statistical properties of fitness landscapes are regarded as the “evolutionary attributes” of biopolymers, such as proteins. Properties such as the number of local peaks and the relative area of the mountainous region to the flat region at the bottom provide insight into the degree of diversity among all possible sequences that must be searched to begin functional evolution, the rate at which a given property evolves, and to what extent an evolutionary process proceeds. These questions are important not only for the design of functional biopolymers by molecular evolutionary engineering but also for experimentally testing scenarios of biopolymer evolution.

**Figure 1:**
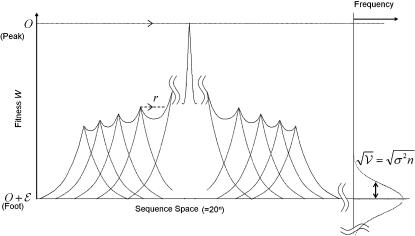
Schematic Representation of *n-k* Fitness Landscape in the Sequence Space. Dashed and solid lines indicate the mean descending slopes of local mountains and ridges between local optima, respectively. Notations of parameters are described in the text.

The *n*-*k* landscape model, in which substitutions occurring on one of *n* sites affect the contribution of residues at *k* other sites to fitness, was proposed as a model of the fitness landscape [2], [3](Figure 1). In this simple model, the only parameters necessary to determine the properties of the fitness landscape, such as ruggedness and frequency of local peaks, are the value of *k* and the difference in altitude between the global peak and the foot, defined as the region in the sequence space where random sequences are located. If *k* = 0, all amino acid sites are independent, and so the effects of substitutions on fitness are additive. As fitness changes gradually with substitutions, the landscape is smooth with a single global peak, which is referred to as a “Mt. Fuji-type” profile. In this case, adaptive walks of the search with single substitutions gradually reach the global peak. On the other hand, larger values of *k* are associated with more rugged landscapes. If substitutions at a single amino acid site affect residues on *k* other sites, the effects of double substitutions on two different sites may not be equal to the sum of the effects of the two independent single substitutions [4]–[7]. Thus, the landscape is rugged with multiple peaks. On a rugged landscape, the adaptive walk can become trapped by local fitness optima. To find the global peak on the rugged landscape, the adaptive walk requires enormous sequence diversity. Therefore, *k* is an essential determinant for the fitness landscape structure. There have been a number of theoretical studies of evolutionary dynamics on both smooth and rugged landscapes [2], [3], [8]–[14]. To obtain insight into the fitness landscapes of proteins, Kauffman and Weinberger applied the *n*-*k* model to affinity maturation of the immunoglobulin V region; based on the number of steps in the adaptive walk up to the local optima, the value of *k* was estimated to be about 40 in this case [2], [3].

Although a great deal is known about the landscape structure near the fitness peaks of native proteins [5], [7], [9], [15], little is known about structures near the bottom, which contain information regarding primordial protein evolution. Experimental molecular evolution from randomly generated polypeptides has been employed to determine how and to what extent a functional protein can evolve according to the principles of Darwinian evolution [16]–[20]. One of the most remarkable findings of these studies is that relatively small degrees of sequence diversity, *e.g.*, 10 different random sequence for esterase activity, are sufficient to allow Darwinian selection of random polypeptides composed of about 140 amino acid residues [17]. Previously, we reported the evolution of phage infectivity with only seven cycles of random mutation and selection of an arbitrarily chosen single random sequence [18]. Infection of *Escherichia coli* by the coliphage fd is mediated by the minor coat protein g3p [21], [22], which consists of three distinct domains connected *via* flexible glycine-rich linker sequences [22]. One of the three domains, D2, located between the N-terminal D1 and C-terminal D3 domains, functions in the absorption of g3p to the tip of the host F-pilus at the initial stage of the infection process [21], [22]. We produced a defective phage, “fd-RP,” by replacing the D2 domain of the fd-tet phage with a soluble random polypeptide, “RP3-42,” consisting of 139 amino acids [23]. The initial defective phage fd-RP showed little infectivity, indicating that the random polypeptide RP3-42 contributes little to infectivity. However, we achieved 240-fold improvement in phage infectivity through seven cycles of random mutagenesis on the replaced polypeptide and selection of the phage clone with the highest infectivity from a library of only about ten mutant phage clones in each generation. The evolvability of arbitrary chosen random sequence suggests that most positions at the bottom of the fitness landscape have routes toward higher fitness.

Although it was shown to be possible for a single arbitrarily chosen polypeptide to evolve infectivity, the evolution stagnated after the 7th generation, which was probably due to the small mutant library size at each generation. Therefore, we have extended *in vitro* molecular evolution by increasing the library size gradually from 10^2^ to 10^6^. By applying the experimental data to an original theory of the adaptive walk on the *n*-*k* fitness landscape model [8], [13], [14], we determined the *k* value and other parameters to plot a protein fitness landscape and discussed its implications regarding the primordial stages of protein evolution and *in vitro* evolutionary molecular engineering.

## Results

### Results of *in vitro* Evolution

**Figure 2:**
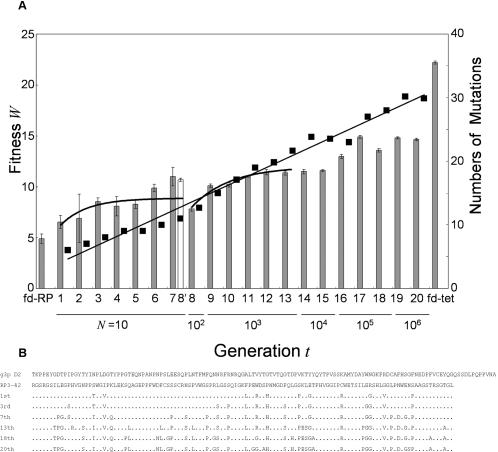
Time Series of Fitness and Amino Acid Sequence in *in vitro* Evolution. (A) Each bar represents the fitness of the fittest mutant in each generation. All generations except the 8′th generation, which was derived from the 7th, are of a single lineage. The error bar is the standard deviation for experiments repeated three times. The fd-tet phage is the wild-type, which possesses the native D2 domain. The solid curves through the 1st-8′th and 8th-13th generations are the theoretical curves fitted with Eqn. (13). For each generation, the average of the synonymous mutations between the initial sequence RP3-42 and selected clones at each generation are shown by the filled squares. (B) The whole sequences of the wild-type D2 domain and the initial random sequence are listed. Substitutions accumulated with generations are indicated in single-amino acid code.

To plot the fitness landscape ranging from the foot to an altitude corresponding to sufficient biological function, we extended our *in vitro* molecular evolution, which we previously carried out up to the 7th generation, with the addition of an enrichment process by which the fittest phage clone(s) becomes dominant through several cycles of infection and growth in *E. coli*. As stagnation occurred due to small library size in our previous experiments, we gradually increased the library size *N* if the time course of infectivity reached a plateau (Figure 2A). For each generation, we prepared a mutant library from the parental population enriched at the previous generation and continued the iterative enrichment process until the increase in infectivity seemed to cease. We used the enriched population as the parental population for the next generation, and the fitness of the parental population at each generation was estimated as the natural logarithm of the infectivity (see Materials and Methods for justification of taking the logarithmic scale as fitness). The infectivity of the evolved clones was increased to 1.7×10^4^-fold as compared to that of fd-RP, which corresponded to an increase in fitness of 9.7. As the library size, *N*, was about 10 until the 7th generation, the fitness stagnated. An increase in *N* to 10^2^ did not increase the fitness significantly to the limit of detection. However, the fitness began to increase with an increase in *N* of 10^3^. By further increasing *N*, we overcame some stagnations in the 20 generations. Before reaching a level comparable to that of wild-type phage, the fitness must increase by 7.6, which requires a huge library size if only substitutions are employed, as discussed below.

No convergence to the wild-type D2 domain was detected. The amino acid sequences of the clones picked randomly from the enriched population showed no significant homology to the wild-type sequence (Figure 2B). Based on detailed analysis of the fitness landscape described below, it is likely that the adaptive walk climbed to a different mountain in the fitness landscape from that where the wild-type sequence exists (see Discussion).

To obtain information regarding the landscape structure from the evolutionary dynamics described above, it is essential to determine the amino acid substitution rate in the mutant library. We determined the DNA sequences corresponding to amino acid residues 12–130 of the random sequence of fd-RP, and this region was subjected to random mutagenesis at each generation. All the clones were subjected to sequence analysis prior to selection until the 7th generation. From these sequences, we estimated the ratio of non-synonymous *vs.* synonymous mutations as 1.8 for our random mutagenesis with error-prone PCR. From the 8th to the 20th generation, we also determined the sequences of the same DNA region for 10 to 16 arbitrarily chosen clones from the selected population at each generation. The number of synonymous mutations after selection accumulated linearly with the number of generations (Figure 2A), and the synonymous mutation rate was estimated to be 1.36. The invariance of the slope indicated that the synonymous mutation rate was affected little by increasing the selection pressure due to the increase in library size from 10 to 10^6^. The non-synonymous mutation rate before selection, *d*, was estimated to be 2.4 by multiplying the synonymous mutation rate by the ratio.

### Determination of the Structure of the *n-k* Landscape

The parameters determining the structure of the *n*-*k* landscape—*O*, the fitness of the global peak; **ε**, the difference in fitness from the foot to the global peak; **ν**, the variance in fitness among all sequences; and *k*, the number of amino acid sites contributions of which to the fitness were influenced by substitutions in other single sites—were determined by analyzing the evolutionary dynamics of the relationship between (1) fitness in the stationary phase and the library size, and (2) the time course of changes in fitness in the transitional phase toward the stationary phase. The method applied here was based on the findings of theoretical studies and its applicability to the experimental data and more detailed analyses are described elsewhere (Aita *et al.*, in preparation).

**Figure 3:**
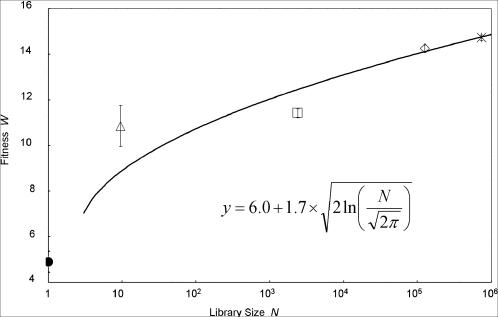
Stationary Values of Fitness against Library Size, *N.* The stationary value of walker's fitness in the stationary phase, *W* ^*^, is given on the ordinate. The fitness at the 0th, the mean fitnesses of the 7th and 8′th, of the 12th and 13th, of the 17th and 18th, and of the 19th and 20th generations are indicated with filled circles, triangles, squares, diamonds, and asterisks, respectively. The library size is shown on the abscissa. We regarded the library size at the initial generation as *N* = 1. The solid curve was drawn with Eqn. (11).

We assumed that the adaptive walk shown in Figure 2A reached stationary phase at the 7th–8′th, 12th–13th, 17th–18th, and 19th–20th generations for library sizes, *N* = 10, 10^3^, 10^5^, and 10^6^, respectively. These stagnations can be explained by the balance between mutation, selection, and random drift due to the limited library size. If a mutant clone with greater fitness than the parent clone appears with a limited library size *N*, it has a high chance of increasing the fitness of the parent clone for the next generation. On the other hand, if most but not all of *N* mutants have lower fitness than the parent clone, one of these mutants can be selected by chance as only a limited number of cells are chosen at random after fitness-dependent growth. Thus, the fitness of the parent clone for the next generation can decrease. Therefore, with a limited library size, *N*, there will be a certain fitness value of the parent clone at stationary phase, given by Eqn. (11) (Materials and Methods) where *d* is the mutation rate (2.4 per generation) and *n* is the length of the amino acid sequence subjected to mutation (119), which corresponds to amino acid residues 12–130 of RP3-42. Note that *n* = 119 implicitly assumes that the region contributes to phage infectivity with little epistatic effect with other regions of the phage genome. Using the mean fitness in the stationary phase for each *N* value in the evolutionary dynamics shown in Figure 2A, we confirmed that the fitness value *W* ^*^ at each stationary phase followed Eqn. (11) (Figure 3). Note that the fitness of the initial defective fd-RP at the 0th generation is plotted at *N* = 1 as the random sequence RP3-42 in fd-RP was assumed to be “selected” among *N* = 1 arbitrarily chosen sequences. This assumption was confirmed by the observation that the infectivity of fd-RP was comparable to that of the deletion mutant phage lacking the D2 domain. Fitting Eqn. (11) to the plot of the *W* ^*^ values against the respective values of *N* (Figure 3), we obtained *O*+**ε** as 6.0 and 4**ν** *n*/*d*(1+*k*) as 5.8. We adopted the estimated values of *W* ^*^ given above, and Eqn. (13) was fitted to the plot of the time course of changes in fitness for the 1st–7th and 8′th generations and for 8th–13th generations, as shown in Figure 2A. This fitting yielded values of *d(*1+*k)/n* = 0.5 for *N* = 10 and 0.39 for *N* = 10^3^, and with *n* = 119 and *d* = 2.4, we estimated the values of *k* as 24 and 18, respectively. Thus, we adopted an average of *k* = 21 for the fitness landscape of phage infectivity. By combining the values determined above and Eqns. (4) and (5) with *k* = 21, we estimated the following parameters: *O* = 21.5, **ε** = −15.5, **ν** = 0.64. It should be noted that the fitness of the wild-type fd-tet phage, which was not used for determination of the parameters, was as high as the global peak.

### Structure of the Fitness Landscape

Based on the *n-k* model with the parameters estimated as described above, we depicted the structure of the fitness landscape of the g3p minor coat protein D2 domain for phage infectivity as follows. First, the frequency of sequences that take the fitness values of *W* obeys the Gaussian distribution given in Eqn. (3) with average *O*+**ε** = 6.0 and variance **ν** = 0.64 (Figure 1). The region where ***W***>*O*+**ε** is above the foot, whereas that where ***W***≤*O*+**ε** is regarded as below the baseline. In the region above the foot, larger fitness *W* of the sequences is associated with a marked reduction in frequency by exp(−(*W*−*O*−**ε**)^2^/2**ν**).

**Figure 4:**
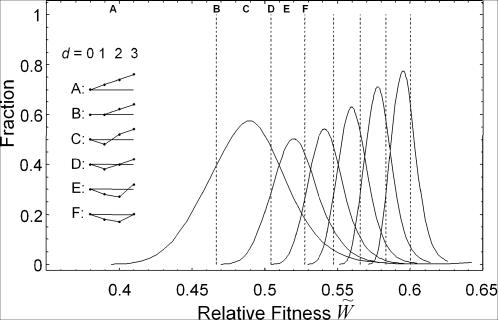
Emergence of Local Optima in the Landscape. Each Gaussian-like curve represents the fraction of local optima in the *r*-th order, 
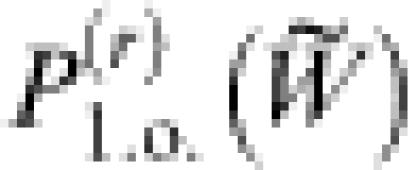
 (*r* = 1, 2, 3, 4, 5, 6). Each dashed line represents the relative fitness where the mutation-selection-random drift balance sets in with all conceivable *d*-fold point mutants of *N* ^(*d*)^_all_ (*d* = 1, 2, 3, 4, 5, 6, 7), given by Eqn. (14). The average local structures at different fitness values are shown schematically.

We found that there are no local optima between the foot and the middle altitude on the landscape, whereas above the middle region the number of local optima is very large. Hereafter, we indicate the altitude on the landscape by the relative fitness, 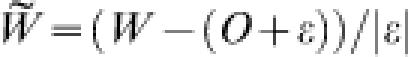; the relative fitnesses at the foot and at the global peak are 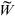 = 0 and 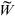 = 1.0, respectively. Figure 4 shows the probability of local optima in the *r*-th order as a function of fitness 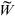. A local optimum in the *r*-th order that takes 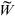 is defined as a sequence in which all conceivable single point mutants, double point mutants,…, and *r*-fold point mutants around it have fitness values lower than 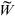 but at least one of *r*+1-fold point mutants has a value greater than 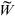. The Gaussian-like curves represent 
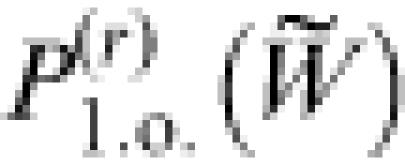
 shown in Eqn. (16) (*r* = 1, 2, 3, 4, 5, 6). The vertical broken lines represent the fitness values, the relative fitness of *W* ^*^ (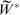), given by substituting *N* with N^(d)^_all_ in Eqn. (11), where the mutation-selection-random drift balance sets when all conceivable *d*-fold point mutants of N^(d)^_all_ (*d* = 1, 2, 3, 4, 5, 6, 7), given by Eqn. (14), are explored in each generation. In the range from 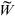 = 0 to 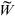 = 0.40, there are so few local optima that the surface on the landscape is smooth. Almost all sequences have at least one fitter mutant among their single point mutants, *i.e.*, they have at least one ascending path. The fraction of the local optima in the first order (*r* = 1) rises above 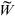 = 0.40 (A in Figure 4). At 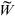 = 0.47 (B in Figure 4), about 40% of sequences belong to local optima in the first order, while 60% of sequences lie on the slopes leading to higher fitness. Around the former sequences, a search with N^(1)^_all_ of all single point mutants will find only lower fitness, whereas around the latter sequences, the search will find at least one with higher fitness. Thus, the adaptive walk with N^(1)^_all_ of all single point mutants will lead to small fluctuations around 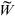 = 0.47 (B in Figure 4). That is, adaptive walks searching a small region of a unit Hamming distance ride on the uneven wave of small local optima, which suggests that there are “neutral paths” [10], [24], [25]. The local optima in the first order (*r* = 1) reach their maximal frequency of 60% at 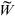 = 0.49 (C in Figure 4). At 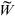 = 0.5 (D in Figure 4), the local optima in the second order, those in the first order, and sequences on slopes leading to higher fitness constitute 30%, 50%, and 20% of all sequences, respectively. On such an uneven surface, adaptive walks with a complete search by N^(2)^_all_ of all double point mutants ride and fluctuate due to the mutation-selection-random drift balance. With further increases in fitness, the local optima tend to have a larger basin size and the frequency of sequences located on the slopes decreases.

## Discussion

We have extended our previous experimental evolution based on phage infectivity, which showed stagnation after the 7th generation. Increasing the library size overcame the stagnations to some extent and resulted in a 1.7×10^4^-fold increase in infectivity as compared to the original phage carrying a random polypeptide of 139 amino acids in place of the D2 domain of the g3p minor coat protein. We applied the fitness data of selected clones at each generation to our theory regarding adaptive walking on the *n*-*k* landscape model to estimate several parameters of the model and calculate the frequencies of local optima depending on their fitness values.

The estimated value of the epistasis parameter *k*, representing the number of amino acid sites the contributions of which to fitness are affected by a single substitution on a given site, was 21 indicating that an arbitrary residue interacts with about 21 amino acid residues through its mutational effects. The *k* value around the level of fitness for native proteins was estimated to be 40 for immunoglobulin [3]. This *k* of 40 is around the native protein on the fitness landscape and is not necessarily close to our value of 21, but may be an overestimate because two assumptions were made in computer simulations to estimate the *k* value: a relatively small population was used, particularly at the beginning, and the cloned immunoglobulins were assumed to be at local peaks. If these assumptions are not true, the estimate of *k* for the native protein will be smaller and may be closer to approximately 20. The interaction of amino acid residues with approximately 20 other residues through mutational effects suggests that a single amino acid residue may interact with 20 other residues through direct or indirect contacts. Clustering the interactions by a size of 20 may allow the clusters to evolve as “modules” [26], [27].

**Figure 5:**
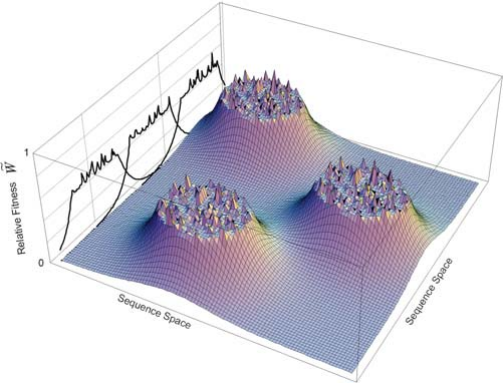
Semi-quantitative Fitness Landscape Plotted from the Experimental Data. The number of sequences decreases with increasing relative fitness. There are many local peaks at fitness values of ≥0.4. Note that the landscape is not shown over a fitness of 0.6 due to the low frequency of the sequence.

The fitness landscape was depicted schematically but semi-quantitatively based on the frequency of local optima calculated from the experimental data (Figure 5). The landscape is smooth from the bottom of the relative fitness 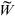 = 0 to 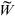 = 0.4. On the other hand, the landscape becomes highly rugged above 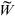 = 0.4. Increasing fitness is associated with the appearance of local optima with larger basins. The ridges between local peaks are composed of sequences with the highest fitness among all possible single point mutants, given by Eqns. (9) and (10) with N^(1)^_all_ For example, a local peak at relative fitness 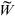 = 0.5 has a neighboring sequence of 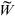 = 0.49 separated by one Hamming distance on its ridge. If the local peak has a basin size of 1, the ridge joins the ridge of another local optimum; if the local peak has a larger basin size of *r*, the ridge declines down to the fitness given by Eqns. (9) and (10) with N^(1)^_all_ recursively *r* times before meeting ridges of other peaks. All ridges meet with others above 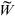 = 0.47, where the mutation-selection-random drift balance sets in with a library size of N^(1)^_all_.

More than one such mountain exists in the fitness landscape of the function for the D2 domain in phage infectivity. The sequence selected finally at the 20th generation has 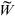 = 0.52 but showed no homology to the wild-type D2 domain, which was located around the fitness of the global peak. The two sequences would show significant homology around 52% if they were located on the same mountain. Therefore, they seem to have climbed up different mountains.

The landscape structure has a number of implications for initial functional evolution of proteins and for molecular evolutionary engineering. First, the smooth surface of the mountainous structure from the foot to at least a relative fitness of 0.4 means that it is possible for most random or primordial sequences to evolve with relative ease up to the middle region of the fitness landscape by adaptive walking with only single substitutions. In fact, in addition to infectivity, we have succeeded in evolving esterase activity from ten arbitrarily chosen initial random sequences [17]. Thus, the primordial functional evolution of proteins may have proceeded from a population with only a small degree of sequence diversity.

Although each sequence at the foot has the potential for evolution, adaptive walking may cease above a relative fitness of 0.4 due to mutation-selection-drift balance or trapping by local optima. It should be noted that the stationary fitness determined by the mutation-selection-drift balance with a library size of N^(*d*)^_all_ is always lower than the fitness at which local optima with a basin size of *d* reach their peak frequencies (Figure 4). This implies that at a given mutation rate of *d*, most adaptive walks will stagnate due to the mutation-selection-drift balance but will hardly be trapped by local optima. Although adaptive walking in our experiment must have encountered local optima with basin sizes of 1, 2, and probably 3, the observed stagnations are likely due only to the mutation-selection-drift balance. Therefore, stagnation was overcome by increasing the library size. In molecular evolutionary engineering, larger library size is generally favorable for reaching higher stationary fitness, while the mutation rate, *d*, may be adjusted to maintain a higher degree of diversity but should not exceed the limit given by *N* = N^(*d*)^_all_ to keep the stationary fitness as high as possible.

In practice, the maximum library size that can be prepared is about 10^13^ [28], [29]. Even with a huge library size, adaptive walking could increase the fitness, 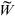, up to only 0.55.

The question remains regarding how large a population is required to reach the fitness of the wild-type phage. The relative fitness of the wild-type phage, or rather the native D2 domain, is almost equivalent to the global peak of the fitness landscape. By extrapolation, we estimated that adaptive walking requires a library size of 10^70^ with 35 substitutions to reach comparable fitness. Such a huge search is impractical and implies that evolution of the wild-type phage must have involved not only random substitutions but also other mechanisms, such as homologous recombination. Recombination among neutral or surviving entities may suppress negative mutations and thus escape from mutation-selection-drift balance. Although the importance of recombination or DNA shuffling has been suggested [30], we did not include such mechanisms for the sake of simplicity. However, the obtained landscape structure is unaffected by the involvement of recombination mutation although it may affect the speed of search in the sequence space.

The exploration of fitness landscapes and the statistical properties of the global or local landscapes are very important issues in the field of *in vitro* molecular evolution. The results of the present study confirmed that our exploration strategy is effective for extracting characteristic properties of the fitness landscape. In addition, we obtained a set of intermediate polypeptides evolving to functional proteins at different stages of the evolutionary trajectory, which will be useful for further studies to investigate the sequence-function relationship through the evolutionary trajectory. We are currently analyzing the results of *in vitro* evolution from the viewpoint of the phylogenic tree that would be expected to provide additional information about the landscape.

## Materials and Methods

### Bacterial Strains and Phage

The *Escherichia coli* strains used in this study were TG1[*supE hsd* *Δ* 5 *thi* *Δ* (*lac-proAB*)/F′ *traD*36 *proAB^+^ lacI* ^q^ *lacZ* *Δ* *M*15], JM109 [*recA*1 *supE*44 *endA*1 *hsdR*17 *gyrA*96 *relA*1 *thi* *Δ*(*lac-proAB*)/F′ *traD*36 *proAB* ^+^ *lacI* ^q^ *lacZM*15], and HB2151 [*Δ*(*lac-proAB*) *ara* *nal* ^r^ *thi*/F′ *traD*36 *proAB* ^+^ *lacI* ^q^ *lacZ* *Δ* *M*15], (Amersham Biosciences Corp., Piscataway, NJ). The fd-tet [31] and fd-RP phage genomes were prepared previously [18]. The fd-1, fd-2, fd-3, fd-4, fd-5, fd-6, and fd-7 phage genomes were selected in each generation in a previous evolutionary study [18].

### Derivation of the Mutant Library Constituting Each Generation

One generation of our evolutionary study consisted of one cycle of mutation and enrichment processes. Random mutations were introduced in the region of the *Sfi*I fragment of the fd-7 genome encoding the target sequence corresponding to the D2 domain of the g3p minor coat protein as follows. The *Sfi*I fragments of phage genomes in the library obtained after the enrichment process of the previous generation were amplified under error-prone PCR conditions [18], [32]. The amplified products were digested with *Sfi*I, and the *Sfi*I fragments were cloned into the corresponding region of the fresh fd-RP vector digested with *Sfi*I. Fresh fd-RP vector is the shorter vector prepared by first digesting the fd-RP DNA with *Bam*HI and ligating the resultant vector fragment, yielding a construct with a truncated *Sfi*I fragment [18]. The resultant derivatives were introduced into *E. coli* JM109 cells by electroporation [33], and the cell suspensions were plated onto 2×YT [34] agar plates containing 40 µg/ml of tetracycline. Cells in the colonies appearing on the plates incubated at 37°C overnight were collected by scraping off in a small volume of 2×YT medium. An equal volume of Luria-Bertani medium [34] containing 30% glycerol was added to the collected cell suspensions, which were then stored at −80°C as the mutant library of the new generation. This mutant library was subjected to the enrichment process described below. Evolution was initiated from the fd-7 genome prepared previously [18]. After the 15th generation, *E. coli* HB2151 was used instead of *E. coli* JM109.

### Preparation of Phage Suspensions of fd-7 and its Derivatives

Aliquots of 10 µl of the mutant library cell suspension described above were dispensed into 10 ml of 2×YT medium containing 20 µg/ml tetracycline and grown at 37°C overnight. The cultures were centrifuged at 6000×*g* for 10 minutes to remove the bacterial cells, and the supernatants containing the phage particles were filtered through Dismic 0.45 µm membranes (Toyo Roshi Kaisha, Ltd., Tokyo, Japan) to ensure the elimination of any remaining bacterial cells. The filtrate containing the phage particles was stored at 4°C as the phage library of the generation for the enrichment process and the phage infectivity assay described below.

### Enrichment Process in Each Generation

The enrichment process consisted of several rounds of phage preparation and infection. The phage particles contained in aliquots of 100 µl of the phage library suspension prepared as described above were allowed to infect freshly grown *E. coli* JM109 cells at OD_600_ 0.8–0.9 (900 µl) for 40 minutes at 37°C. The bacteria-phage mixtures were spread onto 2×YT agar medium containing 40 µg/ml tetracycline by gently swirling the plates. Cells in the colonies grown on the plates after incubation overnight at 37°C were collected as described above and stored at −80°C as the 1st-round enriched library of this generation. The enrichment was repeated until the infectivity of the phage library stopped increasing. Aliquots of 10 µl of the last-round enriched library were dispensed into 10 ml of 2×YT medium containing 20 µg/ml tetracycline and grown at 37°C overnight. The phage genomes in the cells collected by centrifugation of the cultures were purified as the replication form for generating the mutant library in the next generation.

### Phage Infectivity Assay

The number of tetracycline-resistant colonies that grew after infection of *E. coli* with phage particles contained in the library was used as a proxy measure of infectivity, as described previously [18]. To determine the infectivity of each library, the phage particles in the phage suspensions were allowed to infect freshly grown *E. coli* JM109 cells as described previously [18]. The tetracycline-resistant colonies that grew on the plates after overnight incubation at 37°C were counted, and the infectivity of the phage library was expressed as the number of colony forming units per ml of phage suspension (cfu/ml). The infectivity of phage clones and libraries was evaluated in triplicate. Changes in CFU may be due in part to alterations in functions other than infectivity. For example, we found that the release rate of phage clones selected at the 7th generation from *E. coli* cells was about threefold greater than that of fd-RP phage, although the major change in CFU was attributed to the change in infectivity [18]. Therefore, we refer to this CFU value as “infectivity.”

### Estimation of Fitness after Rescaling of Phage Infectivity

Fitness was originally defined as the relative growth rate for predicting population dynamics [35]. The fitness, *W*, was defined here as ln(CFU) under the assumption that CFU is approximately proportional to exp(−*Δ*G/kT), where *Δ*G is the free energy change of phage infection. Thus, *W* can be handled as an apparent energy as described below in the *n*-*k* fitness landscape. Actually, this definition of *W* satisfied some theoretical predictions on the *n*-*k* landscape (Aita *et al.*, in preparation). Experimentally, it has been shown that the mutational effect on protein properties can be described well by taking the logarithmic scale of the equilibrium constant or rate constant [4], [5], [15]. Even for phage infection, it is likely that advantageous mutation increases growth rate exponentially [36], [37].

We used three different strains of *E. coli* for evaluation of infectivity in the selection process: TG1 for generations 0–7, JM109 for generations 8–15, and HB2151 for generations 16–20. For systematic analysis of the lineage through generations 0–20, we used the CFU values for the JM109 strain as the standard measure of infectivity. Then, the infectivity of the best phage clone or clones selected in each of the 0th–20th generations was evaluated simultaneously with the CFU values for JM109. A strong correlation was found between the CFU values for JM109 and those for the other strains, with differences of less than one order of magnitude. The landscape climbed is the infectivity landscape for TG1 strain through generations 0–7 or HB2151 strain through generations 16–20. We converted the time series of the CFU values for TG1 through generations 0–7 or for HB2151 through generations 16–20 to the time series of the CFU values for JM109 as follows:
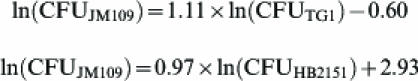
where CFU_XXX_ denotes the CFU value for *E. coli* strain “XXX.” These converted values were then used in the analysis. The fitness, *W*, was defined as ln(CFU_JM109_) for all phages.

### Brief Introduction to the *n*-*k* Fitness Landscape

Let *n* be the number of amino acid residues in a variable region subjected to random mutagenesis through error-prone PCR. The fitness, *W*, for a given amino acid sequence, “α_1_α_2_…α_*n*_,” is defined by:1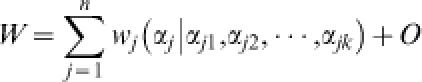 is the “site-fitness,” *i.e.*, the fitness contribution from a particular amino acid residue, *α_j_*, at the *j*-th site when the *k* sites {*j*_1_, *j*_2_,…,*j_k_*} are occupied by particular residues {*α_j_*_1_, *α_j_*_2_,…,*α_jk_*}. The *k* sites {*j*_1_, *j*_2_,…,*j_k_*} are chosen randomly from all *n*-1 sites except the *j*-th site. The site-fitness of an arbitrary amino acid residue, *α* (*e.g.*, *α* = Ala, Cys,…, Tyr), at each site with a given set {*α_j_*_1_, *α_j_*_2_,…,*α_jk_*} is assigned randomly from the following set of 20 values, but degeneracy of assignment is not allowed: 2
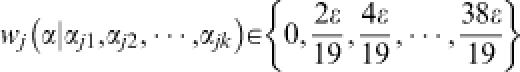



ε(≤0) is a negative constant equivalent to the mean of the site-fitness over all available amino acid residues. Note that there is no significant effect on the theoretical conclusion when the ε value is different by sites [13]. As the site-fitness distribution is given according to the comb-type function, then the variance of the site-fitness *σ* ^2^ is approximately ε^2^/3. As the first term on the right-hand side of Eqn. (1) is ∼0 for the globally optimal sequence (although this is not necessarily guaranteed), the second term *O* is determined as the fitness for the global peak. The fitness landscape resulting from this model is called the “*n*-*k* landscape.” Note that the original *n*-*k* landscape proposed by Kauffman *et al.* is slightly different from the model defined above [2].

Figure 1 shows a schematic representation of the *n*-*k* landscape. **ε**(≤0) is defined as the expectation of the first term in Eqn. (1) and represents the difference in fitness from the peak to the foot of the landscape, where the “foot” is the region in which random sequences are located in the sequence space. Then, *O*+**ε** is the fitness at the foot of the landscape, and corresponds to the expected fitness of an arbitrarily generated random sequence. **ν** is the variance of fitness over all possible sequences in the whole sequence space. The probability density of the fitness over all possible sequences in the whole sequence space approximately follows the Gaussian distribution:3
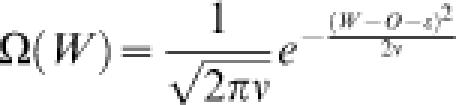

4
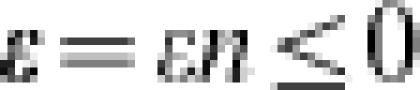

5
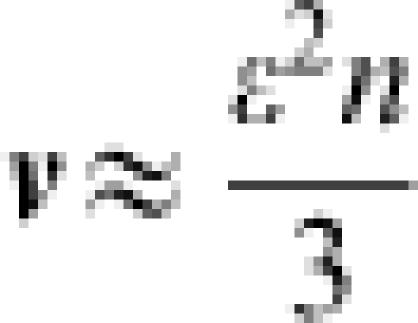



### Summary of our Theory of Evolutionary Dynamics

Here, we present the essence of the theory regarding evolutionary dynamics on the *n*-*k* fitness landscape, with its precise derivation and justification reported elsewhere (Aita *et al.*, in preparation). We consider the following rule of the adaptive walk: the clone(s) with the highest fitness in the previous generation generate *N* mutants in the *t*-th generation, and subsequently the fittest clone(s) among the *N* clones will become new parental clone(s) in the *t*+1-th generation. *N* is the “library size” of mutants to be screened for the next generation. The Hamming distance between a parent and each of its children or mutation rate is *d*. In the *n*-*k* model described above, *d*-fold point substitutions cause changes in site-fitness at about *d*(*k*+1) sites, because a single mutation causes site-fitness changes on the mutated site and *k* other sites. Let *W_t_* be the fitness of the parent in the *t*-th generation. In addition, let *Δ* *W* be the fitness change from the parent to an arbitrary mutant in the mutant population. The probability density of *Δ* *W* with *W_t_* fixed is described by: 6
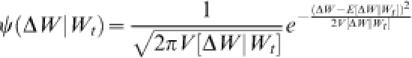
where 7
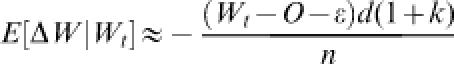

8
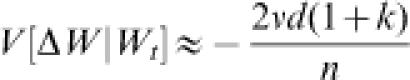



The average and variance of the fitness over the generated mutants were roughly consistent with Eqn. (7) and Eqn. (8), respectively. In addition, the validity of Eqn. (9) was confirmed experimentally. The details are reported elsewhere (Aita *et al.*, in preparation). Using extremal statistics of normal distribution, the expectation of the change in fitness from the parent to a new parent after a single generation is given as follows:9
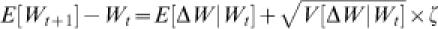
where *ζ* is defined as the expectation of the greatest value among the *N* random numbers from the standard Gaussian probability density, 
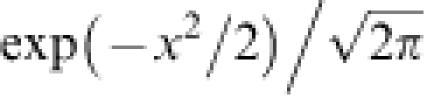
. *ζ* is approximately given by transforming *N via*:10
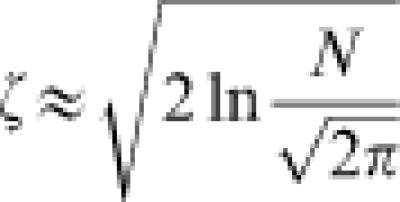



The change in fitness, W*_t_*_+1_−W*_t_*, is designated here as the “evolution rate.” Eqn. (9) indicates that the evolution rate increases with increases in *N*. Substituting Eqns. (7) and (8) into Eqn. (9), we find that, as the adaptive walker climbs the fitness landscape, the evolution rate decreases gradually and finally becomes zero. By solving Eqn. (9) under *E*[*W_t_*_+1_]−*W_t_* = 0, we obtained the fitness value in the stationary phase, *W* ^*^, as follows:11
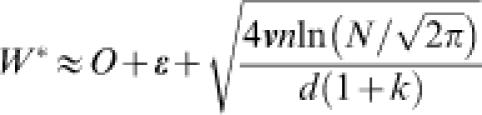



This stationary phase is caused by the mutation-selection-random drift balance [11]. The dependence of the evolution rate on library size, *N*, was confirmed experimentally, as described elsewhere (Aita *et al.*, in preparation).

Using the stationary fitness value, *W* ^*^, Eqn. (9) can be rewritten as follows:12
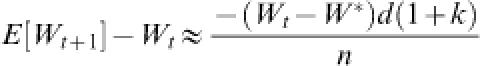



Then, the expectation of the fitness in generation *t* can be obtained approximately by the following function of *t*:13
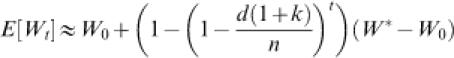
where *W*_0_ is the fitness of the initial sequence.

Next, we refer to the existence of local optima. Let *N* ^(*d*)^_all_ be the number of all conceivable “*d*-fold point mutants,” which are located apart from the parent sequence by the Hamming distance, *d*. *N* ^(*d*)^_all_ is given by:14
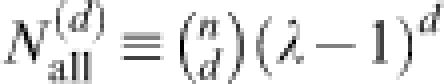
where λ = 20 in this case. Let *W* be the fitness of the parent sequence. From Eqn. (6), the probability that all conceivable *d* -fold point mutants take fitness values less than *W* is given by:15
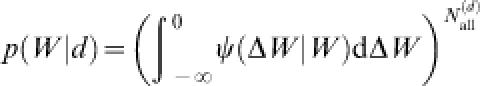



Here, we define the local optima as follows. If all conceivable single point mutants, double point mutants,…, *r*-fold point mutants take fitness values less than *W* but at least one of the *r*+1-fold point mutants takes a fitness value greater than *W*, then the parent sequence is designated the “local optimum in the *r*-th order.” That is, *r* represents the basin size for the local optimum. The probability that a parent with fitness *W* is the local optimum in the *r*-th order is given by:16